# Pediatric congenital buttock sinus tract:10-year experience in a single institution

**DOI:** 10.1186/s12887-019-1806-y

**Published:** 2019-11-06

**Authors:** Kai Wang, Chunhui Peng, Wenbo Pang, Dayong Wang, Tingchong Zhang, Zengmeng Wang, Dongyang Wu, Yajun Chen

**Affiliations:** Department of General Surgery, Beijing Children’s Hospital, Capital Medical University, National Center for Children’s Health, No.56 Nanlishi St, Xicheng District, Beijing, 100045 China

**Keywords:** Pediatric, Buttock sinus tract, Retrorectal cyst, Treatment, Prognosis

## Abstract

**Purpose:**

To review our experience with pediatric congenital buttock sinus tract, and to conclude the clinical characteristics and management of the disease.

**Methods:**

Twenty-two pediatric patients diagnosed with congenital buttock sinus tract were included. Medical records were reviewed, and the patients were followed up. Continuous variables were presented by median and range. Categorical variables were presented as frequencies and percentages.

**Results:**

Among the 22 patients, there were 8 boys (36.4%) and 14 girls (63.6%). The median first onset age was 42 months, and the range was 5 months to 12 years old. Admission age was 69.5 months, with a range from 14 months to 12 years old. Overall prior treatment time was 11 months, ranging from 3 months to 11 years. Twenty-one patients had definite congenital dimples since birth, and later manifested with infection through the dimple. All patients came to the doctor with complaint of the infection. The number of invasive procedures ranged from 0 to 5, with an average of 2. Radiology could exactly display the morphology and show the termination as a retrorectal cyst. The surgical procedure was adopted trans-fistula tract, and the pathological results showed a dermoid cyst in 11 patients and an epidermoid cyst in 10 patients. During the follow-up period of 34.5 months (range, 2 months to 8 years), 19 patients were uneventful and 3 patients suffered recurrence. Two of them underwent a second operation and had no recurrence ever since. The third patient did not receive a second operation, and the refractory infection was still present.

**Conclusions:**

Pediatric congenital buttock sinus tract is rare and has a female predominance in the morbidity. Patients have a distinctive congenital dimple on the buttock with recurrent infection, and there usually exists a congenital sinus tract from the dimple to the retrorectal space. Total excision is the only method for the cure. The nature of the disease is a retrorectal developing dermoid cyst or epidermoid cyst.

## Background

Buttock and perianal infection is common in pediatric patients, especially in the neonatal and infant period [1, 2]. It is usually originated from the epidermis or anal glands, and the most serious condition is the formation of fistula-in-ano. Treatments toward the situation are mainly conservative, including cleaning the local skin and applying external agents, sometimes along with antibiotics; but when fistula forms, surgery is recommended [1, 3], especially for the older patients. Most patients have satisfactory outcomes with appropriate interventions, and few of them will have recurrence.

However, in clinical practice, there is a special group of patients who have refractory, complex and recurrent buttock and perianal infection, and usually with sinus tract formation [4–7]. They have an older onset age and usually have distinctive congenital dimples for the infection. The etiology, clinical manifestations, and treatments are totally different. However, it has rarely been reported, and the clinical characteristics, treatments, and prognosis of such patients remain to be revealed.

The purpose of this study is to retrospectively review our experience with pediatric congenital buttock sinus tract over the past 10 years and to conclude the clinical characteristics and management of the disease, in order to provide a better understanding of this special disease.

## Methods

### Patients

Twenty-two pediatric patients who were diagnosed with congenital buttock sinus tract, admitted to the General Surgery Department of our hospital, underwent surgical procedures between January 2009 and April 2019, and had available follow-up data were included in this study. The medical records of these patients were retrospectively reviewed with attention to the first onset age, admission age, overall prior treatment time, gender, clinical manifestations, site and number of congenital dimples, radiology, history of invasive procedures, surgical procedures, and pathology of sinus tract samples. All patients were followed up through telephone contact and the outpatient service.

### Statistical analysis

Continuous variables were presented by median and range. Categorical variables were presented as frequencies and percentages.

## Results

**Fig. 1:**
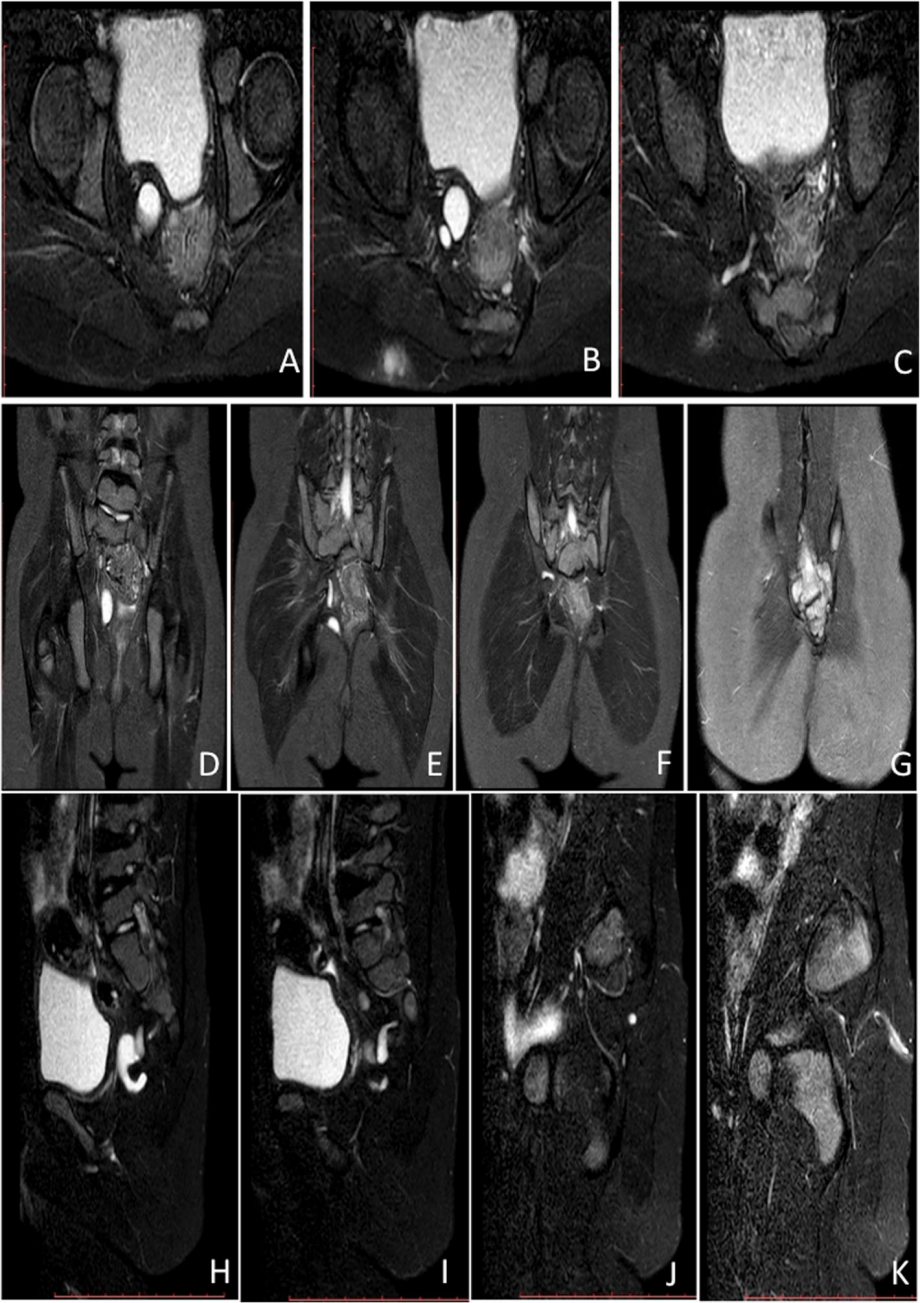
Axial (**a-c**), coronal (**d-g**), and sagittal (**h-k**) MRI on T2WI showed a sinus tract from the dimple to the retrorectal space, and the termination was a cyst

Pediatric congenital buttock sinus tract was diagnosed in 22 patients over the past 10 years in our hospital, including 8 boys (36.4%) and 14 girls (63.6%); the male to female ratio was 1:1.75. The median first onset age was 42 months, and the range was 5 months to 12 years old. Admission age at our hospital was 69.5 months, with a range from 14 months to 12 years old. Overall prior treatment time was 11 months, ranging from 3 months to 11 years. Among the 22 patients (Table 1), 21 had definite congenital dimples; 3 patients had two dimples on one buttock and postanal area, and the remaining 18 had only one dimple on either side of the buttock or postanal area. All patients came to the doctor with the complaint of recurrent buttock infection through the dimple, and thus most of them had been treated several times as perianal infection or fistula-in-ano. The history of invasive procedures included puncture of pus, incision and drainage, and excision of the fistula, and the number of invasive procedures ranged from 0 to 5, with an average of 2 times. One patient (Number 22) who had two dimples, one on the left buttock and another at the postanal area, was diagnosed with a duplication of the rectum and underwent a surgical excision in another hospital, while the sinus tract was not completely excised. The pathological result showed a dermoid cyst, rather than a duplication of the rectum, and she had recurrent buttock infection soon after that surgery. Magnetic resonance imaging (MRI) could exactly display the morphology of buttock sinus tract and show the termination, which was always a cyst lying in the retrorectal space (Fig. 1). However, recurrent buttock infection was the only symptom, none of the patients had constipation or difficult defecation, and the cyst was impalpable via digital examination of rectum (DER). Of all 22 patients, only one had a rib malformation, scoliosis, and horseshoe kidney; and no patient had sacrum or anorectal malformation.

**Table 1:** General condition of the 22 patients

No.	Gender	Number of dimples	Site of dimples	First onset age (M)	Overall prior treatment time (M)	Number of invasive procedures	Termination of the sinus tract	Pathology	Follow-up time (M)	Recurrence after surgery	Combined malformations
1	F	1	Left buttock	84	14	1	Retrorectal space	Epidermoid cyst	115	N	N
2	F	1	Right buttock	72	9	2	Retrorectal space	Epidermoid cyst	111	Y	N
3	M	1	Left buttock	24	8	0	Retrorectal space	Epidermoid cyst	110	N	N
4	F	1	Postanal area	48	22	1	Retrorectal space	Epidermoid cyst	105	N	N
5	F	1	Right buttock	72	75	1	Retrorectal space	Epidermoid cyst	98	N	N
6	M	1	Left buttock	5	9	2	Retrorectal space	Epidermoid cyst	84	N	N
7	F	1	Postanal area	12	141	2	Retrorectal space	Dermoid cyst	82	N	N
8	F	1	Left buttock	36	12	1	Retrorectal space	Dermoid cyst	56	N	N
9	M	2	Right buttock and postanal area	108	3	0	Retrorectal space	Dermoid cyst	50	N	N
10	F	1	Left buttock	36	5	0	Left ilium	Dermoid cyst	39	N	N
11	M	1	Right buttock	85	7	1	Retrorectal space	Dermoid cyst	37	N	N
12	M	1	Right buttock	48	4	1	Retrorectal space	Dermoid cyst	32	N	N
13	M	1	Right buttock	12	36	1	Retrorectal space	Dermoid cyst	32	N	N
14	F	0	–	36	33	4	Retrorectal space	Epidermoid cyst	30	Y	N
15	M	1	Left buttock	12	21	5	Retrorectal space	Epidermoid cyst	27	N	N
16	F	2	Left buttock and postanal area	48	5	0	Retrorectal space	Epidermoid cyst	27	N	N
17	F	1	Postanal area	84	8	1	Retrorectal space	Epidermoid cyst	25	N	N
18	F	1	Left buttock	6	51	3	Retrorectal space	Dermoid cyst	22	N	N
19	F	1	Right buttock	144	5	1	Retrorectal space	Dermoid cyst	20	Y	Rib malformation, scoliosis, horseshoe kidney
20	M	1	Left buttock	12	32	0	Left ilium	Dermoid cyst	16	N	N
21	F	1	Left buttock	12	85	0	Left ilium	Dermoid cyst	14	N	N
22	F	2	Left buttock and postanal area	120	10	3	Retrorectal space	Dermoid cyst	9	N	N

**Fig. 2:**
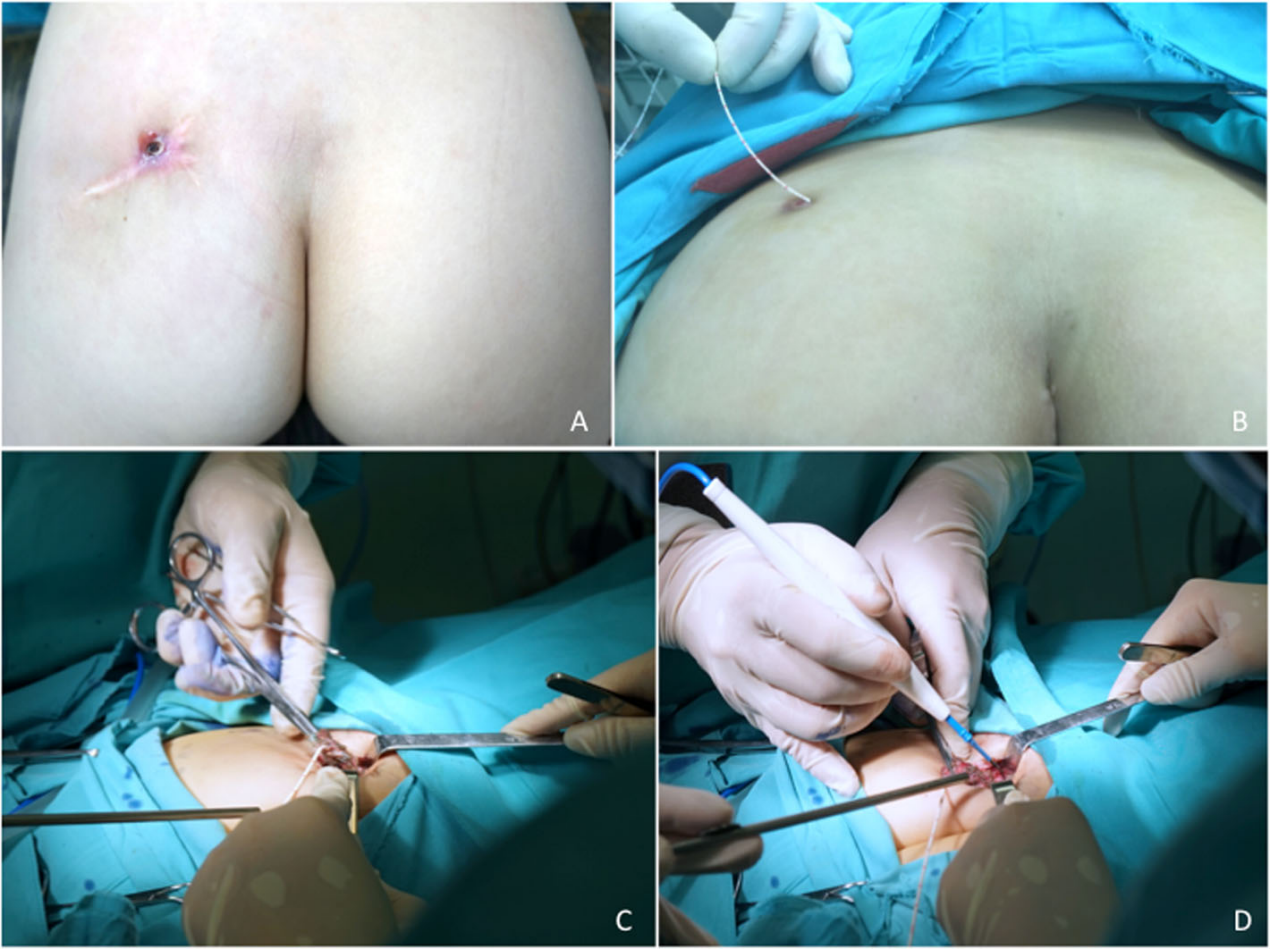
Surgical procedures. The patient was placed in a prone position to expose the dimple (**a**), and we inserted the ureteral catheter through the dimple to the sinus tract and injected methylene blue (**b**). Dissection should be close to the tract layer-by-layer (**c-d**) until the retrorectal space is reached

**Fig. 3:**
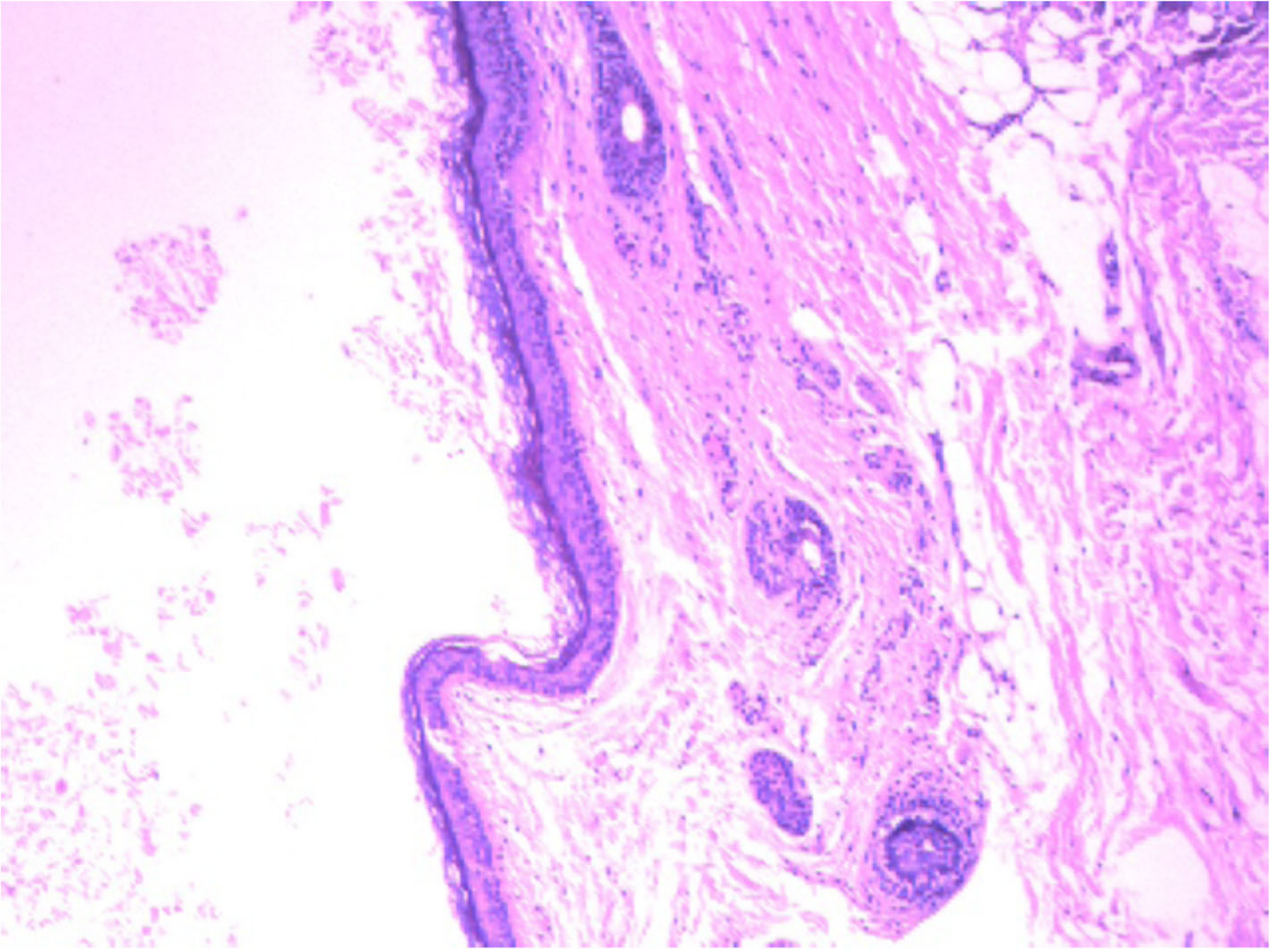
The pathology of the congenital sinus tract was a retrorectal dermoid cyst (HE × 4) that comprised squamous epithelium and epithelial appendages

The congenital dimple was always the external orifice of the fistula (Fig. 2a). The surgical procedure (Fig. 2b-d) we adopted was trans-fistula tract, and the patient was placed in either a prone or a horizontal position depending on whether the site of the dimple was on the buttock or postanal area. We injected methylene blue for a better display of the sinus tract before surgery. Dissection of the sinus tract showed that the fistula tract always extended from the dimple to the retrorectal space, and the terminal part of the tract was enlarged as a cyst, containing hair and sebum. Among the 22 patients, 19 had retrorectal termination and 3 patients terminated at the left ilium. The three patients who had two dimples showed both connections with the retrorectal cyst. The pathology showed a dermoid cyst in 12 patients (Fig. 3) and an epidermoid cyst in 10 patients.

During the follow-up period of 34.5 months (range, 2 months to 8 years) after surgery in our hospital, 19 patients had a satisfactory postoperative course, and 3 patients suffered recurrence approximately 3 to 6 months after surgery. One patient (Number 19) underwent a second operation 3 months later and had an uneventful postoperative course since then. Another patient (Number 2) had recurrence 6 months after the first operation and underwent a second operation with coccyx excision for better exposure. The retrorectal cyst was difficult for completely excision, and thus cyst wall cauterization was adopted; the patient had no recurrence ever since. The third patient (Number 14) did not receive a second operation, and the refractory and recurrent buttock and perianal infection was still a serious issue bothering her childhood.

## Discussion

The etiology, clinical characteristics, management and prognosis of pediatric congenital buttock sinus tract are rarely documented in the literature, and this is the first consecutive case study up to the present.

After retrospectively analyzing the clinical data, we found that congenital buttock sinus tract had an obvious female predominance in the morbidity; the male to female ratio was 1:1.75. Most of the patients had recurrence and complex buttock and perianal infection; invasive treatment was performed several times with no cure, and the situation was always treated as fistula-in-ano. The overall prior treatment time was 11 months. The reasons for an inaccurate diagnosis may be as follows. First, compared with the common fistula-in-ano, pediatric congenital buttock sinus tract has a relatively low morbidity. The clinical manifestations are extremely similar and nonspecific [4]; both of the diseases could manifest as buttock and postanal redness and swelling, ulceration and suppuration, without difficult in defecation or constipation. Besides, a few patients with fistula-in-ano also had recurrence after appropriate management, due to unfavorable nursing and hygiene habits. However, unlike fistula-in-ano, which has a male predominance [1, 3], congenital buttock sinus tract has a female predominance. Second, compared with rectum duplication which exhibits a congenital dimple-like external orifice in the postanal area and may have defecation excrete or early infection, pediatric congenital buttock sinus tract has dimple without infection at the beginning, and latter turned on infection through the dimple around 42 months of age; and the postanal area dimple has lower incidence than the buttock dimple in pediatric congenital buttock sinus tract. Third, different from currarino syndrome which has retrorectal neoplasm, sacrum and anorectal malformation, pediatric congenital buttock sinus tract patients have no sacrum or anorectal malformation, and the retrorectal neoplasm is always dermoid or epidermoid cyst, with no difficult in defecation, and the cyst is impalpable through DER. Exact diagnosis is of great significance, which include a congenital dimple on the buttock and/or postanal area [5, 6] since birth, latter turned on infection through the dimple including, no difficult in defecation, and no sacrum or anorectal malformation. In addition, radiology results, especially MRI, contribute greatly to the accurate diagnosis, as it may disclose a definite retrorectal cyst connected to the dimple through a sinus tract.

The pathological results of the specific disease were divided into dermoid cyst and epidermoid cyst depending on whether or not it contained skin appendages, such as hair follicles, sebaceous and sweat glands. Thus, the nature of congenital buttock sinus tract is a retrorectal developing cyst, including a dermoid cyst or epidermoid cyst. The retrorectal space [7], which is bordered superiorly by the peritoneal reflection, inferiorly by the supralevator muscle complex, anteriorly by the rectum, posteriorly by Waldeyer’s presacral fascia and laterally by the iliac vessels, is an area in which multiple residual embryological developments occur, including the ectoderm, hindgut, neural elements, bone and connective tissues [8, 9]. The estimated incidence of retrorectal tumors is 1 in 40,000 admissions; among them, 66% are congenital, including a retrorectal developing dermoid or epidermoid cyst [7, 10]. Other tumor types, including neurological cysts, tailgut cysts [11, 12], cystic rectal duplication and teratomas, have lower incidences. Developing dermoid or epidermoid cyst is one of the retrorectal embryonic remnant tumors that results from the defective closure of the ectodermal tube [7]. Moreover, dimple on the buttock is also the result of an ectoderm remnant from incomplete embryonic development, and it is always communicated to the developing cyst through a sinus tract; hence, it could be explained that the dimple, the sinus tract, and the developing cyst are the result of congenital defective closure of the ectodermal tube. And the buttock dimple could be a distinctive mark of the disease [13].

Different from fistula-in-ano, conservative treatment or incision and drainage has a limited effect on the outcome, and all patients will immediately relapse without total excision as in this study. Total excision is the only method for a radical cure, and unlike other retrorectal tumors, better exposure could be gained via the trans-fistula approach for congenital buttock sinus tract. In our experience, when the patient has a buttock dimple, with or without a postanal dimple, the prone position should be chosen. However, if there is only one dimple located in the postanal area, the patients should be placed horizontally with the feet hanged up. We insert the ureteral catheter through the dimple to the sinus tract and inject methylene blue to display the morphology of the sinus tract before surgery (Fig. 2b). All these efforts are aimed for a better exposure and visualization. The sinus tract always extends to the retrorectal space, and when breakage occurs during surgery, hair and white sebum is always witnessed. The key point of the surgery, and the avoiding of recurrence, is the complete excision of the sinus tract and retrorectal cyst. Any residual epithelium of the sinus tract or retrorectal cyst will affirmatively recall a recurrence. As the three patients with recurrent infection in the study, they would surely need a second operation for the total excision of the sinus tract and retrorectal cyst (like patient Number 19), or at least for the destruction of the residual epithelium (like patient Number 2); otherwise, there was no radical cure (like patient Number 14).

In terms of the retrorectal cyst excision, some professors suggested coccygectomy in cases of recurrence [8, 14] and for better exposure; however, we do not consider this a necessity. First, coccygectomy has no correlation with the recurrence of pediatric congenital buttock sinus tract. In this study, we concluded definitely that the nature of the retrorectal developing cyst is either a dermoid cyst or an epidermoid cyst, both of which are benign neoplasms. Different from teratomas, the recurrence has no correlation with the coccyx. Recurrence mainly depends on the incomplete excision of the sinus tract and retrorectal cyst, with residual epithelium. Second, Losanoff suggested that the removal of retrorectal lesions with no attachment to the sacrum was not difficult and the surrounding structures could be spared, so coccygectomy was not necessary [15]. Only when the exposure becomes difficult, coccygectomy could be considered; as in case Number 2, the exposure in the second operation turned out to be more difficult due to the disorganization and adhesion of local tissues, and thus coccygectomy was adopted. Therefore, coccygectomy is not a necessity in the surgery unless the exposure is extremely difficult.

Although pediatric congenital buttock sinus tract patients may have recurrence, once the total excision is taken, a radical cure is on the way. This study showed that, after the total excision of the sinus tract and retrorectal cyst, the infection would have no recurrence, and the patients would lead a normal childhood.

## Conclusions

Pediatric congenital buttock sinus tract is rare and has a female predominance in the morbidity. It is usually mistreated as fistula-in-ano. Patients have a distinctive congenital dimple on the buttock with recurrent infection, and there usually exists a congenital sinus tract from the dimple to the retrorectal space. Total excision of the sinus tract and the retrorectal cyst is the only method for radical cure. The nature of the disease is a retrorectal developing dermoid or epidermoid cyst.

## Data Availability

The data is available from the corresponding author on reasonable request.

## Abbreviations

DER: Digital examination of rectum
MRI: Magnetic resonance imaging
